# Off to a Good Start: The Early Development of the Neural Substrates Underlying Visual Working Memory

**DOI:** 10.3389/fnsys.2016.00068

**Published:** 2016-08-18

**Authors:** Allison Fitch, Hayley Smith, Sylvia B. Guillory, Zsuzsa Kaldy

**Affiliations:** Department of Psychology, University of Massachusetts BostonBoston, MA, USA

**Keywords:** visual working memory, frontoparietal network, ventral stream, early development, neonatal lesions in primates, infants, preschoolers

## Abstract

Current neuroscientific models describe the functional neural architecture of visual working memory (VWM) as an interaction of the frontal-parietal control network and more posterior areas in the ventral visual stream (Jonides et al., 2008; D'Esposito and Postle, 2015; Eriksson et al., 2015). These models are primarily based on adult neuroimaging studies. However, VWM undergoes significant development in infancy and early childhood, and the goal of this mini-review is to examine how recent findings from neuroscientific studies of early VWM development can be reconciled with this model. We surveyed 29 recent empirical reports that present neuroimaging findings in infants, toddlers, and preschoolers (using EEG, fNIRS, rs-fMRI) and neonatal lesion studies in non-human primates. We conclude that (1) both the frontal-parietal control network and the posterior cortical storage areas are active from early infancy; (2) this system undergoes focalization and some reorganization during early development; (3) and the MTL plays a significant role in this process as well. Motivated by both theoretical and methodological considerations, we offer some recommendations for future directions for the field.

## Introduction

Working memory is a limited-capacity system for the maintenance and manipulation of information in service of ongoing tasks. The classic model of working memory (WM, Baddeley and Hitch, 1974) distinguishes the central executive system and two different sensory buffers for the temporary storage of visual and auditory information (an additional system, the episodic buffer, was later added: Baddeley, 1986). This multicomponent model has framed essentially all research on WM for more than 20 years. More recent “state-based” WM models (Cowan, 1988; Oberauer, 2002; McElree, 2006), however, question basic assumptions of the multicomponent model, claiming that there are no separate WM-specific storage systems in the brain; instead, representations held in WM are temporarily activated long-term memory (LTM) representations. According to this view, storage of sensory information involves posterior cortices; visual WM (VWM) representations, for example, have been localized in various stages of the ventral stream, starting in the occipital cortex (Harrison and Tong, 2009; Serences et al., 2009) and continuing to inferior temporal cortex (Miller et al., 1991). Maintenance and manipulation of WM representations (the functions of the central executive) depend upon a frontal-parietal network (Awh and Jonides, 2001; Curtis and D'Esposito, 2003), in particular, anterior insula, lateral prefrontal cortex (PFC), dorsal anterior cingulate cortex, and areas within and surrounding the intraparietal sulcus (Seeley et al., 2007).

This conceptualization of WM is grounded in an extensive body of neuroscientific research, the majority of which has been conducted with human adults (for reviews, see Jonides et al., 2008; D'Esposito and Postle, 2015; Eriksson et al., 2015). WM undergoes significant postnatal development, with far-reaching consequences on cognitive development in general (Bull et al., 2008). Behavioral studies have shown that the ability to hold information in VWM emerges in infancy (Káldy and Leslie, 2003, 2005; Ross-Sheehy et al., 2003; Zosh and Feigenson, 2012), and gradually improves throughout childhood (Riggs et al., 2006; Cowan et al., 2010; Simmering, 2012) and adolescence (Isbell et al., 2015). It is outside of the scope of this mini-review to provide a comprehensive overview of the entire behavioral literature (see Kibbe, 2015; Cowan, 2016; Reynolds and Romano, 2016, in this *Research Topic*); instead, we will examine whether recent findings from neuroscientific studies of early VWM development can be fit into the adult model above.

We limit our focus to studies that examine VWM in the first 5 years of life. While there is an abundant fMRI literature on children older than 6–7 years of age (e.g., Geier et al., 2009; von Allmen et al., 2014), this method currently cannot be used with very young children, and here we focus on what is known about these mechanisms before this age. The studies reviewed here employ a variety of neurophysiological methods (primarily electroencephalography, EEG, and functional Near-Infrared Spectroscopy, fNIRS) in human infants and young children (Table 1) and lesions in young primates (Table 2).

**Table 1:** **Summary of findings in the early neurodevelopment of VWM in humans**.

**Method**	**Authors and year**	**VWM behavioral task**	**Response modality**	**infant (0–2)**	**Early childhood (3–5)**	**Adult comparison**	**Age**	**Cross-sectional vs. longitudinal**	**What needs to be encoded**	**Number of items to encode**	**VWM-related activity changes**
EEG, HR	Bell and Wolfe, 2007	A-not-B; EF tasks	Looking; verbal response	Yes	Yes		8 months to 4.5 years	L	Location	1	Both EEG power and coherence became more localized with age to frontal and fronto-temporal and fronto-occipital connections
EEG, HR	Cuevas and Bell, 2011	A-not-B	Looking	Yes			5–10 months	L	Location	1	EEG power increased with age in all four lobes, coherence only in frontal connections
EEG, HR	Cuevas et al., 2012a	A-not-B	Looking	Yes			5–10 months	L	Location	1	Increase in EEG coherence becomes more localized with age to the fronto-parietal connections
EEG	Cuevas et al., 2012b	A-not-B	Looking	Yes			8 months	One	Location	1	Higher EEG coherence in frontal and fronto-parietal regions for correct responses
EEG, HR	Cuevas et al., 2012c	A-not-B	Looking	Yes			10 months	One	Location	1	Higher fronto-temporal coherence when inhibition/updating is required
EEG, HR	Bell, 2012	A-not-B	Looking	yes			8 months	One	Location	1	Fronto-parietal coherence higher for correct responses
fNIRS	Baird et al., 2002	DR	Reaching	Yes			5–12 months	L	Existence	1	Increased bilateral frontal activity correlates with success in object maintenance
EEG	Kaufman et al., 2003	VoE	Looking	Yes			6 months	One	Existence	1	Higher gamma-band power during object maintenance in right temporal cortex
EEG	Kaufman et al., 2005	VoE	Looking	Yes			6 months	One	Existence	1	
EEG	Leung et al., 2016	VoE	Looking	Yes			7 months	One	Number	1, 2	1 vs. 2 objects: higher gamma-band activity in right occipital cortex
fNIRS	Wilcox et al., 2005	VoE	Looking	Yes			6.5 month	One	Shape	1	Increased anterior temporal activity when infant notices featural change
fNIRS	Wilcox et al., 2008	VoE	Looking	yes			6.5 month	One	multifeature, Shape, color	1	
fNIRS	Wilcox et al., 2009	VoE	Looking	Yes			6.5 months	One	multifeature	1	
fNIRS	Wilcox et al., 2010	VoE	Looking	Yes			6 months	One	Shape, color; spatiotemp.	1	Increased activity in temporal cortex during featural changes, in parietal cortex during spatiotemp. changes
fNIRS	Wilcox et al., 2012	VoE	Looking	Yes			5, 12 months	C	Shape, color	1	Posterior temporal activity decreases with age. Increased occipital activity during all object maintenance tasks
fNIRS	Wilcox et al., 2014	VoE	Looking	Yes			8 months	One	Shape, color	1	
fNIRS	Wilcox and Biondi, 2016	VoE	Looking	Yes			5, 8, 12 months	C	Shape, color	1	
DTI	Short et al., 2013	A-not-B	Reaching	Yes			12 months	One	Location	1	Maturity of bilateral frontal, fronto-temporal, and certain thalamo-cortical connections correlate with performance
rs-fMRI	Alcauter et al., 2014	A-not-B	Reaching	Yes			0–24 month	L	Location	1	Thalamus-salience network connectivity predicted performance
fNIRS	Buss et al., 2014	Change detection	Verbal response		Yes		3, 4 years	C	Shape	1, 2, 3	Load-dependent activity in posterior parietal and frontal cortices, more robust parietal activity with age
fNIRS	Perlman et al., 2016	DR	Reaching		Yes		3–7 years	C	Location	1	Lateral prefrontal activity increased with age and delay
fNIRS	Tsujimoto et al., 2004	Change detection	Manual response		Yes	Yes	5.5 years, adult	One	Location	2	Higher lateral prefrontal activity during task
fNIRS	Tsujii et al., 2009	Change detection	Manual response		Yes		5–7 years	L	Location	2	Prefrontal activity during task becomes more right lateralized with age. Lateralization change correlates with performance

*EEG, electroencephalography; HR, heart rate; rs-fMRI, resting state functional magnetic resonance imaging; DTI, Diffusion Tensor Imaging; fNIRS, functional near-infrared spectroscopy; A-not-B, A-not-B task; DR, Delayed Response; VoE, Violation of Expectation task; L, longitudinal; C, cross-sectional; “one,” only one age group tested*.

**Table 2:** **Summary of findings in the early neurodevelopment of VWM in non-human primates**.

**Method**	**Authors and Year**	**VWM behavioral task**	**Response modality**	**infant (0–2)**	**Early childhood (3–5)**	**Adult comparison**	**Age**	**Cross-sectional vs. longitudinal**	**What needs to be encoded**	**Number of items to encode**	**Lesion-induced VWM performance changes**
Lesion	Heuer and Bachevalier, 2011	SU-DNMS, Obj-SO	Reaching			Yes	Neo-HC adults	One	Featural	1, 3	Object maintenance with updating is not affected in Neo-HC adults, but serial order monitoring is impaired
Lesion	Heuer and Bachevalier, 2013	SOMT	Reaching			Yes	Neo-HC adults	One	Serial order	3, 4	
DTI	Meng et al., 2014	SOMT	Reaching			Yes	Neo-HC adults	One	Serial order	see H&B, 2013	Left ventromedial prefrontal cortex integrity correlated with performance on the monitoring task in Neo-HC adults
rs-fMRI	Meng et al., 2016	SOMT	Reaching			Yes	Neo-HC adults	One	Serial order	3, 4	Reduced dlPFC connectivity with V4 and IT predicted poorer performance
Lesion	Glavis-Bloom et al., 2013	Foraging Task	Reaching			Yes	Neo-HC adults	One	Feature and location combined	7	Neo-HC adults were impaired in both object/location maintenance tasks
Lesion	Weiss et al., 2016	SU-DNMS, Obj-SO, SOMT	Reaching			Yes	Neo-PRh adults	One	Featural, serial order	1, 3, 4	Object maintenance with updating is impaired in Neo-PRh adults, but serial order monitoring is preserved

*DTI, Diffusion Tensor Imaging; SU-DNMS, session-unique Delayed Nonmatch-to-Sample; SOMT, Serial Order Memory Test; Obj-SO, Object Self-Order task; Neo-HC adults, adult macaques who received neurotoxic hippocampal lesions during the first 2 weeks of life; Neo-PRh adults, adult macaques who received neurotoxic lesions in the perirhinal region during the first 2 weeks of life; L, longitudinal; C, cross-sectional; “one,” only one age group tested*.

Structural and functional brain development progresses in parallel. Both classic brain anatomical studies in synaptic density (Huttenlocher and Dabholkar, 1997) and more recent structural connectivity studies using DTI (Qiu et al., 2015) found a posterior-to-anterior progression during the first few years of life, with white matter developing in the occipital and temporal cortices before frontal areas. While our focus in this mini-review is on the *functional* development of the system underlying VWM, we will also discuss a few groundbreaking studies where researchers were able to link behavioral performance in a VWM task with myelination of a specific network (Short et al., 2013; Meng et al., 2014).

## Neurodevelopment of the human VWM system: infancy (0–2 years)

Many of the neuroimaging studies examining infant VWM development employed the classic A-not-B task in conjunction with optical imaging (fNIRS) or EEG. In this task, an object is hidden at one of two locations and the infant is allowed to manually search for it. Once the infant repeatedly succeeds at one location, the object is then hidden at the other location. In the looking-based version of this task, looking times to the two locations are contrasted.

In one of the first studies to measure regional blood-flow changes in infants using fNIRS, Baird et al. (2002) found that prefrontal cortex (PFC) activity increased with success on an object maintenance task. More recently, EEG power and coherence measures from the entire scalp have been used to examine VWM task-related and age-related changes in the frontal-parietal network of infants (Bell and Wolfe, 2007; Cuevas and Bell, 2011; Bell, 2012; Cuevas et al., 2012a,b,c). Cuevas et al. (2012a), for example, found that frontal EEG power and heart rate predicted VWM performance in infants at 10 months, but not at 5 months. In another study, successful performance on the A-not-B task was found to be related to increased frontal-parietal coherence at 8 months (Bell, 2012; Cuevas et al., 2012b). These findings suggest that the frontal-parietal network supports successful VWM performance between 8 and 10 months.

During the infancy period, functional connectivity of the VWM network appears to become less diffuse with age. Cuevas et al. (2012a) found an increase in EEG coherence relative to baseline across the entire scalp in 5-month-olds but only between the medial frontal and occipital electrode sites in 10-month-olds. This finding is additionally supported by the observation of increased focalization of frontal-parietal network activity between 8 months and 4.5 years of age, which may reflect more efficient communication (Bell and Wolfe, 2007).

Resting-state fMRI (rs-fMRI) has been used to identify functional connections between brain regions in the absence of any task. This latter aspect makes this method particularly attractive for studies of early development, as infants can be scanned during sleep. In a pioneering study, Alcauter et al. (2014) tracked the development of resting-state networks in infants from birth to 2 years of age and their VWM performance. In addition to significant gains in synchrony among prefrontal and parietal regions at age one, it was found that connectivity between the thalamus and the salience network (which includes the insula, the cingulate, and frontal cortices, and is considered a sub-network of the frontal-parietal network in adults, see Elton and Gao, 2014) at age one predicted VWM performance at age two. In a DTI tractography study, the same group found that myelination of the tracts connecting frontal and parietal cortices predicted VWM performance in 1-year-old infants (Short et al., 2013). These studies thus corroborate the EEG findings that frontal-parietal connectivity is present before the end of the first year, and is related to VWM development. However, because salience network activity is functionally dissociated from WM performance in adults (Seeley et al., 2007; Elton and Gao, 2014), it is likely this network undergoes functional reorganization between toddlerhood and adulthood.

The involvement of posterior cortical areas in infant VWM has primarily been examined using more modern behavioral paradigms, such as Violation-of-Expectation (VoE), in conjunction with fNIRS, or EEG. Using fNIRS, Wilcox and colleagues found that the anterior temporal cortex showed consistent activation when infants noticed a change in the features of an object that they held in mind when it reappeared from behind an occluder (thus, this feature change “violated” their expectations; Wilcox et al., 2005, 2008, 2009, 2010, 2014). Task-related activation in the posterior temporal cortex gradually decreased from 5 to 12 months, and the occipital cortex was active during all object maintenance tasks. This decrease in activation in posterior temporal cortex may reflect functional reorganization of object processing areas over the course of development (Wilcox et al., 2012, 2014; Wilcox and Biondi, 2016). Converging evidence for maintenance related activity in posterior storage areas has been reported by Kaufman et al. (2003, 2005) using EEG. They found that increased gamma-band (20–60 Hz) activity in the right temporal cortex of 6-month-olds was associated with the maintenance of object representations behind an occluder (Kaufman et al., 2003, 2005). More recently, Kaufman and colleagues showed that the same response was higher in the right occipital cortex when infants kept two vs. one object in VWM (Leung et al., 2016). This result raises the possibility of finding a load-dependent neural signature of information storage in infant VWM.

In sum, the literature concerning the neural substrates of VWM systems in infants points toward an early emerging frontal-parietal network; one that is present and active even before age one (Bell, Cuevas; connectivity studies). Studies by Wilcox, Kaufman and their colleagues found storage-related VWM activity in the temporal and occipital cortices as well, which may mirror similar findings in adults in the ventral visual stream (for a recent review, see Lee and Baker, 2016).

## Neurodevelopment of the human VWM system: early childhood (3–5 years)

To date, only a handful of neuroimaging studies have examined VWM during the early childhood period, and all used fNIRS. The lack of neuroimaging (both structural and functional) conducted with this notoriously challenging age range is primarily due to practical limitations: Preschool-age children require special experimental designs as they are rarely willing to participate for an extended time, and they often do not follow verbal instructions reliably. One notable limitation of three of the four fNIRS studies reviewed below is that hemodynamic responses were measured only in the frontal areas (or in Buss et al., 2014, in the frontal and the parietal cortices). Thus, conclusions were necessarily constrained to these regions.

Tsujimoto et al. (2004) found that lateral PFC activity in 5.5-year-old children was very similar to adults' during a change detection task: One of the most widely used paradigms in adult VWM research, participants are briefly presented with a set of to-be-remembered items, and following a short delay are tested on whether or not the items have changed (Pashler, 1988; Luck and Vogel, 1997). Using the same task with a small longitudinal sample, Tsujii et al. (2009) found that between 5 and 7 years of age, increased VWM performance correlated with right lateralization of frontal activity.

More recently, Buss et al. (2014) found that the frontal-parietal network was active in 3- and 4-year-olds during a change detection task, where load was systematically manipulated. Overall, they demonstrated greater involvement of parietal cortical areas relative to frontal areas, as well as increased parietal activity in 4-year-olds relative to 3-year-olds. Prior studies found that, in adults, activity in the parietal cortex was load-dependent for small set sizes, and leveled off at the behaviorally-defined capacity limit (Todd and Marois, 2004; Palva et al., 2011). In 3- and 4-year-olds this activity was load-dependent, but continued to increase beyond the observed capacity limit—a finding that warrants further investigation. In a similar investigation of delay-dependent activity, Perlman et al. (2016) manipulated the length of delays (2 vs. 6 s) and found age-dependent activation in lateral PFC in children between 3 and 7 years of age, and that children recruited this area more during longer delays. As the ventrolateral PFC is involved in maintenance, this finding suggests increased active rehearsal of information with age.

In sum, it appears that the frontal-parietal network becomes increasingly adult-like throughout early childhood. Increased recruitment of prefrontal and parietal areas point to increased focalization of the frontal-parietal system, while increased lateralization to the right hemisphere suggests adult-like specialization of this network for visuospatial tasks (Thomason et al., 2009). Because recordings were not made from the temporal and occipital areas, at the current time we cannot draw any conclusions about the involvement of the posterior cortices. The paucity of research in this age range creates a gap in our understanding of the development of VWM.

## Neurodevelopment of the non-human primate VWM system: effects of neonatal lesions

Both the frontal-parietal network and the posterior storage areas (e.g., IT) have multiple connections to the medial temporal lobe (MTL; Lavenex et al., 2002). While most current neuroscientific methods used in young children (fNIRS, EEG) do not allow access to these deep structures, primate lesion studies have provided a wealth of findings about the role of these structures in early development. Unlike adult lesion studies, which can only provide information about the relative contribution of a brain structure in a fully-formed system, neonatal lesion studies have the advantage of examining the downstream effects of a lesion on the *developing* system^1^. In the following section, we will focus on the role of the MTL in the development of the frontal-parietal network.

Heuer and Bachevalier (2011) examined the contribution of the hippocampus to the development of VWM abilities. Here they utilized a delayed response task (also widely used in classic behavioral studies with infants; e.g., Diamond and Doar, 1989), where participants are presented with one object (the sample), followed by a delay, and then a choice between a matching object and a non-matching object. In the delayed-non-match-to-sample (DNMS) version of this task, participants are rewarded for selecting the non-matching object. Results showed that adult macaques that received neonatal hippocampal lesions (henceforth: Neo-HC) performed as well as sham-operates on a DNMS task (requires maintenance and putatively relies on the vlPFC, see Petrides, 1995). However, these macaques failed to even meet training criterion on an object self-ordered pointing task (Obj-SO) in which participants selected baited food wells in a different order on successive trials (requires manipulation, specifically, monitoring serial order, and putatively relies on the dlPFC; Petrides, 1995).

Follow-up studies using other dlPFC-associated VWM tasks have provided supporting evidence: Neo-HC macaques made significantly more errors than controls on a serial-order memory (SOMT) task (Heuer and Bachevalier, 2013), and in a foraging task were more likely than controls to return to boxes they had already visited, especially if that box previously contained the animal's preferred food (Glavis-Bloom et al., 2013). Thus early hippocampal lesions lead to deficits in VWM manipulation, but not in maintenance. The finding that early hippocampal damage leads to deficits on a task that taps dlPFC has been replicated in human patients who suffered hypoxic-ischaemic events early in life (Geva et al., 2016).

In addition to hippocampal lesions, Weiss et al. (2016) found that neonatal lesions to another area of MTL, the perirhinal cortex, impacted VWM performance on tasks believed to rely on the vlPFC. In their study, macaques with neonatal lesions of perirhinal cortex (Neo-PRh) were impaired on a DNMS task at short delays, as well as an Obj-SO task; both repeated stimuli across trials, and thus required trial-to-trial updating of information in VWM. In contrast, Neo-PRh animals performed well on a task that used novel stimuli across trials (SOMT), thus did not require updating, suggesting that the perirhinal cortex is not involved in manipulation of WM contents *per se*, but rather interference resolution or associated executive functions (e.g., inhibition).

These findings suggest that the MTL gives rise to the development of PFC-associated VWM skills, such as manipulation and interference resolution, likely through reciprocal neuroanatomical connections (Goldman-Rakic et al., 1984; Aggleton et al., 2015). Two recent connectivity studies provide converging evidence for this. Early hippocampal damage led to both reduced white matter (Meng et al., 2014) and decreased resting-state connectivity (Meng et al., 2016) between the dlPFC and the medial PFC and several posterior areas, such as IT and V4, in adult macaques. These anatomical and functional impairments correlated with poorer performance on the SOMT (Meng et al., 2014, 2016). This correlation underscores the importance of the hippocampus, as well as the frontal-parietal network in the development of VWM abilities: By adulthood, Neo-HC macaques had not developed compensatory mechanisms for VWM. This stands in stark contrast to a similar neonatal lesion study demonstrating compensatory mechanisms for rule learning and recognition memory following lesions to the vlPFC (Malkova et al., 2016).

## Summary and future directions

The goal of this mini-review was to examine the neurophysiological evidence regarding the early emergence of the VWM network that involves both the frontal-parietal control network and the posterior storage areas that have been identified in adults. Our first conclusion is that both of these systems seem to be active from as early as the second half of the first year in humans.

A handful of longitudinal and cross-sectional studies reviewed here point to a gradual focalization of the frontal-parietal system throughout development (see the works of Bell and her colleagues). We also see some evidence for the functional reorganization of the network during the early life period: for example, a shift away from the salience network from infancy to adulthood (Alcauter et al., 2014), and increasing reliance on the parietal cortex during the preschool years (Buss et al., 2014). These changes may reflect a specialization within the network. Furthermore, findings from non-human primates have demonstrated the significance of the medial temporal lobe in the development of the lateral PFC (see the works of Bachevalier and her colleagues).

Related to the emergence of posterior information storage areas, a number of studies found object-maintenance related activity in both occipital and temporal lobes in infancy (see the works of Kaufman and his colleagues). Studies on VWM mechanisms in early childhood have not recorded from these posterior areas, so our understanding of how these areas support VWM in this age range is, at the moment, limited.

A defining characteristic of VWM is its limited capacity. This functional characteristic can serve as a signature to identify VWM storage-related mechanisms: In these structures, activity is expected to gradually increase with the number of to-be-remembered items, and then remain constant when capacity limit has been reached (e.g., Palva et al., 2011). In studies that aim to find this signature, the adult cognitive neuroscience literature has adopted a useful psychophysical measure to quantify VWM capacity (Cowan's *k*^2^). Research on school-age children has recently begun to examine how memory load affects the recruitment of different parts of the VWM system using this measure (e.g., Shimi et al., 2014; Kharitonova et al., 2015). Importantly, this approach has already been applied successfully in preschoolers (Buss et al., 2014, reviewed above).

Based on both theoretical and methodological considerations, the ideal design to study neurodevelopmental change in the VWM system has the following attributes:

- Uses the same behavioral task across ages from infants to adults (or at least between infants and young children or young children and older ones).
- Quantifies capacity behaviorally (e.g., with Cowan's *k*), and measures neural activity as a function of capacity, in order to identify storage-related mechanisms.
- Uses a longitudinal sample, to minimize the effects of inter-individual variability.

Some of the studies to date have two of these features, but none have all three. Because of its versatility and low task demands, the change detection paradigm is the best positioned to meet criterion (a) in the near future. Thus, a crucial open question for future studies is how neural activity in the VWM network changes in children under 3 years of age using this task. As well, future studies with preschool-age children that record from posterior cortices (using whole-brain nets, see e.g., Sato et al., 2012) should elucidate the role of these structures in VWM beyond infancy.

Despite all the methodological challenges that are involved in studying brain functions in infants, young children, and young primates, research on early VWM neurodevelopment has gotten off to an exciting start. Several different physiological methods have already yielded converging results, and recent advances in neuroimaging methods (e.g., Cutini and Brigadoi, 2014; Graham et al., 2015), will likely lead to an expansion of research in the near future. We look forward to an exciting period in the study of the early developmental unfolding of the VWM system.

## Author contributions

AF and HS contributed equally to this manuscript by selecting and summarizing relevant studies and writing multiple sections of this review. SG contributed significantly to the section on EEG and rs-fMRI studies. ZK has developed the theoretical perspective with the help of AF and HS. All four authors contributed to the writing and editing of the paper.

## Funding

The authors were supported by National Institutes of Health's grant R15HD086658 and a Seed Grant from the Simons Foundation under the auspices of the Simons Center for the Social Brain at MIT (#319294) awarded to ZK.

### Conflict of Interest Statement

The authors declare that the research was conducted in the absence of any commercial or financial relationships that could be construed as a potential conflict of interest.

## Footnotes

^1^The earliest neuroscientific studies of the development of the frontal cortex used these techniques as well (Goldman, 1971; Miller et al., 1973), and demonstrated the role of both the dorsolateral and the ventrolateral PFC (dlPFC and vlPFC) in VWM. By connecting findings in PFC-lesioned macaques and human infants, Diamond and Goldman-Rakic (1989) laid the one of the first building blocks of developmental cognitive neuroscience.

^3^The formula is *k* = N ^*^ (H + CR − 1), where N is the number of items presented, H is hit rate, CR is correct rejection rate (Cowan et al., 2005).

## Abbreviations

DR: delayed response
DNMS: delayed non-match to sample
DTI: diffusion tensor imaging
EEG: electroencephalography
fNIRS: functional near infrared spectroscopy
fMRI: functional magnetic resonance imaging
HR: heart rate
dlPFC: dorsolateral prefrontal cortex
LTM: long-term memory
MTL: medial temporal lobe
Neo-HC: neonatally lesioned in the hippocampus
Neo-PRh: neonatally lesioned in the perirhinal cortex
Obj-SO: object self-ordered pointing task
PRh: perirhinal cortex
rs-fMRI: resting state fMRI
SOMT: Serial Order Memory Task
VoE: Violation of Expectation
vlPFC: ventrolateral prefrontal cortex
VWM: visual working memory
WM: working memory.

## References

[B1] Aggleton J. P. Wright N. F. Rosene D. L. Saunders R. C. (2015). Complementary patterns of direct amygdala and hippocampal projections to the macaque prefrontal cortex. Cereb. Cortex 25, 4351–4373. 10.1093/cercor/bhv019 25715284 PMC4612443

[B2] Alcauter S. Lin W. Smith J. K. Short S. J. Goldman B. D. Reznick J. S.. (2014). Development of thalamocortical connectivity during infancy and its cognitive correlations. J. Neurosci. 34, 9067–9075. 10.1523/JNEUROSCI.0796-14.2014 24990927 PMC4078084

[B3] Awh E. Jonides J. (2001). Overlapping mechanisms of attention and spatial working memory. Trends Cogn. Sci. 5, 119–126. 10.1016/S1364-6613(00)01593-X 11239812

[B4] Baddeley A. D. (1986). Working Memory. Oxford Psychology Series #11. Oxford: Clarendon Press.

[B5] Baddeley A. D. Hitch G. (1974). Working memory, in *The Psychology of Learning and Motivation*, Vol. 8, ed Bower G. H. (New York, NY: Academic Press), 47–89.

[B6] Baird A. A. Kagan J. Gaudette T. Walz K. A. Hershlag N. Boas D. A. (2002). Frontal lobe activation during object permanence: data from near-infrared spectroscopy. NeuroImage 16, 1120–1126. 12202098 10.1006/nimg.2002.1170

[B7] Bell M. A. (2012). A psychobiological perspective on working memory performance at 8 months of age: psychobiology of infant working memory. Child Dev. 83, 251–265. 10.1111/j.1467-8624.2011.01684.x 22103396

[B8] Bell M. A. Wolfe C. D. (2007). Brain reorganization from infancy to early childhood: Evidence from EEG power and coherence during working memory tasks. Dev. Neuropsychol. 31, 21–38. 10.1080/87565640709336885 17305436

[B9] Bull R. Espy K. A. Wiebe S. A. (2008). Short-term memory, working memory, and executive functioning in preschoolers: longitudinal predictors of mathematical achievement at age 7 years. Dev. Neuropsychol. 33, 205–228. 10.1080/87565640801982312 18473197 PMC2729141

[B10] Buss A. T. Fox N. Boas D. A. Spencer J. P. (2014). Probing the early development of visual working memory capacity with functional near-infrared spectroscopy. Neuroimage 85, 314–325. 10.1016/j.neuroimage.2013.05.034 23707803 PMC3859697

[B11] Cowan N. (1988). Evolving conceptions of memory storage, selective attention, and their mutual constraints within the human information processing system. Psychol. Bull. 104, 163–191. 3054993 10.1037/0033-2909.104.2.163

[B12] Cowan N. (2016). Working memory maturation: can we get at the essence of cognitive growth? Perspect. Psychol. Sci. 11, 239–264. 10.1177/1745691615621279 26993277 PMC4800832

[B13] Cowan N. Elliott E. M. Scott Saults J. Morey C. C. Mattox S. Hismjatullina A.. (2005). On the capacity of attention: its estimation and its role in working memory and cognitive aptitudes. Cogn. Psychol. 51, 42–100. 10.1016/j.cogpsych.2004.12.001 16039935 PMC2673732

[B14] Cowan N. Morey C. C. AuBuchon A. M. Zwilling C. E. Gilchrist A. L. (2010). Seven-year-olds allocate attention like adults unless working memory is overloaded: capacity and attention allocation. Dev. Sci. 13, 120–133. 10.1111/j.1467-7687.2009.00864.x 20121868 PMC2819460

[B15] Cuevas K. Bell M. A. (2011). EEG and ECG from 5 to 10 months of age: developmental changes in baseline activation and cognitive processing during a working memory task. Int. J. Psychophysiol. 80, 119–128. 10.1016/j.ijpsycho.2011.02.009 21338632 PMC3096566

[B16] Cuevas K. Bell M. A. Marcovitch S. Calkins S. D. (2012a). Electroencephalogram and heart rate measures of working memory at 5 and 10 months of age. Dev. Psychol. 48, 907–917. 10.1037/a0026448 22148943 PMC3560404

[B17] Cuevas K. Raj V. Bell M. A. (2012b). Functional connectivity and infant spatial working memory: a frequency band analysis: infant frequency bands and EEG coherence. Psychophysiology 49, 271–280. 10.1111/j.1469-8986.2011.01304.x 22092263 PMC3262932

[B18] Cuevas K. Swingler M. M. Bell M. A. Marcovitch S. Calkins S. D. (2012c). Measures of frontal functioning and the emergence of inhibitory control processes at 10 months of age. Dev. Cogn. Neurosci. 2, 235–243. 10.1016/j.dcn.2012.01.002 22483073 PMC3322365

[B19] Curtis C. E. D'Esposito M. (2003). Persistent activity in the prefrontal cortex during working memory. Trends Cogn. Sci. 7, 415–423. 10.1016/S1364-6613(03)00197-9 12963473

[B20] Cutini S. Brigadoi S. (2014). Unleashing the future potential of functional near-infrared spectroscopy in brain sciences. J. Neurosci. Methods 232, 152–156. 10.1016/j.jneumeth.2014.05.024 24880046

[B21] D'Esposito M. Postle B. R. (2015). The cognitive neuroscience of working memory. Ann. Rev. Psychol. 66, 115–142. 10.1146/annurev-psych-010814-015031 25251486 PMC4374359

[B22] Diamond A. Doar B. (1989). The performance of human infants on a measure of frontal cortex function, the delayed response task. Dev. Psychobiol. 22, 271–294. 2707496 10.1002/dev.420220307

[B23] Diamond A. Goldman-Rakic P. S. (1989). Comparison of human infants and rhesus monkeys on Piaget's AB task: evidence for dependence on dorsolateral prefrontal cortex. Exp. Brain Res. 74, 24–40. 2924839 10.1007/BF00248277

[B24] Elton A. Gao W. (2014). Divergent task-dependent functional connectivity of executive control and salience networks. Cortex 51, 56–66. 10.1016/j.cortex.2013.10.012 24315034

[B25] Eriksson J. Vogel E. K. Lansner A. Bergström F. Nyberg L. (2015). Neurocognitive architecture of working memory. Neuron 88, 33–46. 10.1016/j.neuron.2015.09.020 26447571 PMC4605545

[B26] Geier C. F. Garver K. Terwilliger R. Luna B. (2009). Development of working memory maintenance. J. Neurophysiol. 101, 84–99. 10.1152/jn.90562.2008 18971297 PMC2637004

[B27] Geva S. Cooper J. M. Gadian D. G. Mishkin M. Vargha-Khadem F. (2016). Impairment on a self-ordered working memory task in patients with early-acquired hippocampal atrophy. Dev. Cogn. Neurosci. 20, 12–22. 10.1016/j.dcn.2016.06.001 27288821 PMC4973808

[B28] Glavis-Bloom C. Alvarado M. C. Bachevalier J. (2013). Neonatal hippocampal damage impairs specific food/place associations in adult macaques. Behav. Neurosci. 127, 9–22. 10.1037/a0031498 23398438 PMC3736558

[B29] Goldman P. S. (1971). Functional development of the prefrontal cortex in early life and the problem of neuronal plasticity. Exp. Neurol. 32, 366–387. 4999861 10.1016/0014-4886(71)90005-7

[B30] Goldman-Rakic P. S. Selemon L. D. Schwartz M. L. (1984). Dual pathways connecting the dorsolateral prefrontal cortex with the hippocampal formation and parahippocampal cortex in the rhesus monkey. Neuroscience 12, 719–743. 6472617 10.1016/0306-4522(84)90166-0

[B31] Graham A. M. Pfeifer J. H. Fisher P. A. Lin W. Gao W. Fair D. A. (2015). The potential of infant fMRI research and the study of early life stress as a promising exemplar. Dev. Cogn. Neurosci. 12, 12–39. 10.1016/j.dcn.2014.09.005 25459874 PMC4385461

[B32] Harrison S. A. Tong F. (2009). Decoding reveals the contents of visual working memory in early visual areas. Nature 458, 632–635. 10.1038/nature07832 19225460 PMC2709809

[B33] Heuer E. Bachevalier J. (2011). Neonatal hippocampal lesions in rhesus macaques alter the monitoring, but not maintenance, of information in working memory. Behav. Neurosci. 125, 859–870. 10.1037/a0025541 21928873 PMC3226899

[B34] Heuer E. Bachevalier J. (2013). Working memory for temporal order is impaired after selective neonatal hippocampal lesions in adult rhesus macaques. Behav. Brain Res. 239, 55–62. 10.1016/j.bbr.2012.10.043 23137699 PMC3804023

[B35] Huttenlocher P. R. Dabholkar A. S. (1997). Regional differences in synaptogenesis in human cerebral cortex. J. Comp. Neurol. 387, 167–178. 9336221 10.1002/(sici)1096-9861(19971020)387:2<167::aid-cne1>3.0.co;2-z

[B36] Isbell E. Fukuda K. Neville H. J. Vogel E. K. (2015). Visual working memory continues to develop through adolescence. Front. Psychol. 6:696. 10.3389/fpsyg.2015.00696 26074849 PMC4443298

[B37] Jonides J. Lewis R. L. Nee D. E. Lustig C. A. Berman M. G. Moore K. S. (2008). The mind and brain of short-term memory. Annu. Rev. Psychol. 59, 193–224. 10.1146/annurev.psych.59.103006.093615 17854286 PMC3971378

[B38] Káldy Z. Leslie A. (2005). A memory span of one? Object identification in 6.5-month-old infants. Cognition 57, 153–177. 10.1016/j.cognition.2004.09.009 16226561

[B39] Káldy Z. Leslie A. M. (2003). Identification of objects in 9-month-old infants: integrating ‘what’ and ‘where’ information. Dev. Sci. 6, 360–373. 10.1111/1467-7687.00290

[B40] Kaufman J. Csibra G. Johnson M. H. (2003). Representing occluded objects in the human infant brain. Proc. Roy. Soc. B Biol. Sci. 270, S140–S143. 10.1098/rsbl.2003.0067 14667363 PMC1809955

[B41] Kaufman J. Csibra G. Johnson M. H. (2005). Oscillatory activity in the infant brain reflects object maintenance. Proc. Natl. Acad. Sci. U.S.A. 102, 15271–15274. 10.1073/pnas.0507626102 16230640 PMC1257741

[B42] Kharitonova M. Winter W. Sheridan M. A. (2015). As working memory grows: a developmental account of neural bases of working memory capacity in 5- to 8-year old children and adults. J. Cogn. Neurosci. 27, 1775–1788. 10.1162/jocn_a_00824 25961641

[B43] Kibbe M. M. (2015). Varieties of visual working memory representation in infancy and beyond. Curr. Dir. Psychol. Sci. 24, 433–439. 10.1177/0963721415605831

[B44] Lavenex P. Suzuki W. A. Amaral D. G. (2002). Perirhinal and parahippocampal cortices of the macaque monkey: projections to the neocortex. J. Comp. Neurol. 447, 394–420. 10.1002/cne.10243 11992524

[B45] Lee S. H. Baker C. I. (2016). Multi-voxel decoding and the topography of maintained information during visual working memory. Front. Syst. Neurosci. 10:2. 10.3389/fnsys.2016.00002 26912997 PMC4753308

[B46] Leung S. Mareschal D. Rowsell R. Simpson D. Iaria L. Grbic A.. (2016). Oscillatory activity in the infant brain and the representation of small numbers. Front. Syst. Neurosci. 10:4. 10.3389/fnsys.2016.00004 26903821 PMC4744938

[B47] Luck S. J. Vogel E. K. (1997). The capacity of visual working memory for features and conjunctions. Nature 390, 279–281. 9384378 10.1038/36846

[B48] Malkova L. Alvarado M. C. Bachevalier J. (2016). Effects of separate or combined neonatal damage to the orbitofrontal cortex and the inferior convexivity on object recognition in monkeys. Cereb. Cortex 26, 618–627. 10.1093/cercor/bhu227 25260702 PMC4712798

[B49] McElree B. (2006). Accessing recent events, in The Psychology of Learning and Motivation, Vol. 46, ed Ross B. H.(San Diego, CA: Academic Press), 155–200.

[B50] Meng Y. Hu X. Bachevalier J. Zhang X. (2016). Decreased functional connectivity in dorsolateral prefrontal cortical networks in adult macaques with neonatal hippocampal lesions: Relations to visual working memory deficits. Neurobiol. Learn. Mem.. 10.1016/j.nlm.2016.04.003 [Epub ahead of print]. 27063864 PMC6329462

[B51] Meng Y. Payne C. Li L. Hu X. Zhang X. Bachevalier J. (2014). Alterations of hippocampal projections in adult macaques with neonatal hippocampal lesions: a diffusion tensor imaging study. NeuroImage 102, 828–837. 10.1016/j.neuroimage.2014.08.059 25204865 PMC4252575

[B52] Miller E. A. Goldman P. S. Rosvold H. E. (1973). Delayed recovery of function following orbital prefrontal lesions in infant monkeys. Science 182, 304–306. 4200506 10.1126/science.182.4109.304

[B53] Miller E. K. Li L. Desimone R. (1991). A neural mechanism for working and recognition memory in inferior temporal cortex. Science 254, 1377–1379. 1962197 10.1126/science.1962197

[B54] Oberauer K. (2002). Access to information in working memory: exploring the focus of attention. J. Exp. Psychol. Learn. Mem. Cogn. 28, 411–421. 12018494

[B55] Palva S. Kulashekhar S. Hämäläinen M. Palva J. M. (2011). Localization of cortical phase and amplitude dynamics during visual working memory encoding and retention. J. Neurosci. 31, 5013–5025. 10.1523/JNEUROSCI.5592-10.2011 21451039 PMC3083635

[B56] Pashler H. (1988). Familiarity and visual change detection. Percept. Psychophys. 44, 369–378. 3226885 10.3758/bf03210419

[B57] Perlman S. B. Huppert T. J. Luna B. (2016). Functional near-infrared spectroscopy evidence for development of prefrontal engagement in working memory in early through middle childhood. Cereb. Cortex 26, 2790–2799. 10.1093/cercor/bhv139 26115660 PMC4869813

[B58] Petrides M. (1995). Impairments on nonspatial self-ordered and externally ordered working memory tasks after lesions of the mid-dorsal part of the lateral frontal cortex in the monkey. J. Neurosci. 15, 359–375. 7823141 10.1523/JNEUROSCI.15-01-00359.1995 PMC6578311

[B59] Qiu A. Mori S. Miller M. I. (2015). Diffusion tensor imaging for understanding brain development in early life. Ann. Rev. Psychol. 66, 853–876. 10.1146/annurev-psych-010814-015340 25559117 PMC4474038

[B60] Reynolds G. D. Romano A. C. (2016). The development of attention systems and working memory in infancy. Front. Syst. Neurosci. 10:15. 10.3389/fnsys.2016.00015 26973473 PMC4776056

[B61] Riggs K. J. McTaggart J. Simpson A. Freeman R. P. (2006). Changes in the capacity of visual working memory in 5- to 10-year-olds. J. Exp. Child Psychol. 95, 18–26. 10.1016/j.jecp.2006.03.009 16678845

[B62] Ross-Sheehy S. Oakes L. M. Luck S. J. (2003). The development of visual short-term memory capacity in infants. Child Dev. 74, 1807–1822. 10.1046/j.1467-8624.2003.00639.x 14669897

[B63] Sato H. Hirabayashi Y. Tsubokura H. Kanai M. Ashida T. Konishi I.. (2012). Cerebral hemodynamics in newborn infants exposed to speech sounds: a whole-head optical topography study. Hum. Brain Mapp. 33, 2092–2103. 10.1002/hbm.21350 21714036 PMC6870359

[B64] Seeley W. W. Menon V. Schatzberg A. F. Keller J. Glover G. H. Kenna H.. (2007). Dissociable intrinsic connectivity networks for salience processing and executive control. J. Neurosci. 27, 2349–2356. 10.1523/JNEUROSCI.5587-06.2007 17329432 PMC2680293

[B65] Serences J. T. Ester E. Vogel E. Awh E. (2009). Stimulus-specific delay activity in human primary visual cortex. Psychol. Sci. 20, 207–214. 10.1111/j.1467-9280.2009.02276.x 19170936 PMC2875116

[B66] Shimi A. Kuo B. C. Astle D. E. Nobre A. C. Scerif G. (2014). Age group and individual differences in attentional orienting dissociate neural mechanisms of encoding and maintenance in visual STM. J. Cogn. Neurosci. 26, 864–877. 10.1162/jocn_a_00526 24236697

[B67] Short S. J. Elison J. T. Goldman B. D. Styner M. Gu H. Connelly M.. (2013). Associations between white matter microstructure and infants' working memory. NeuroImage 64, 156–166. 10.1016/j.neuroimage.2012.09.021 22989623 PMC3838303

[B68] Simmering V. R. (2012). The development of visual working memory capacity during early childhood. J. Exp. Child Psychol. 111, 695–707. 10.1016/j.jecp.2011.10.007 22099167

[B69] Thomason M. E. Race E. Burrows B. Whitfield-Gabrieli S. Glover G. H. Gabrieli J. D. E. (2009). Development of spatial and verbal working memory capacity in the human brain. J. Cogn. Neurosci. 21, 316–332. 10.1162/jocn.2008.21028 18510448 PMC2746557

[B70] Todd J. J. Marois R. (2004). Capacity limit of visual short-term memory in human posterior parietal cortex. Nature 428, 751–754. 10.1038/nature02466 15085133

[B71] Tsujii T. Yamamoto E. Masuda S. Watanabe S. (2009). Longitudinal study of spatial working memory development in young children. NeuroReport 20, 759–763. 10.1097/WNR.0b013e32832aa975 19352206

[B72] Tsujimoto S. Yamamoto T. Kawaguchi H. Koizumi H. Sawaguchi T. (2004). Prefrontal cortical activation associated with working memory in adults and preschool children: an event-related optical topography study. Cereb Cortex 14, 703–712. 10.1093/cercor/bhh030 15084489

[B73] von Allmen D. Y. Wurmitzer K. Klaver P. (2014). Hippocampal and posterior parietal contributions to developmental increases in visual short-term memory capacity. Cortex 59, 95–102. 10.1016/j.cortex.2014.07.010 25151641

[B74] Weiss A. R. Nadji R. Bachevalier J. (2016). Neonatal perirhinal lesions in rhesus macaques alter performance on working memory tasks with high proactive interference. Front. Syst. Neurosci. 9:179. 10.3389/fnsys.2015.00179 26778978 PMC4700260

[B75] Wilcox T. Biondi M. (2016). Functional activation in the ventral object processing pathway during the first year. Front. Syst. Neurosci. 9:180. 10.3389/fnsys.2015.00180 26778979 PMC4700261

[B76] Wilcox T. Bortfeld H. Woods R. Wruck E. Armstrong J. Boas D. (2009). Hemodynamic changes in the infant cortex during the processing of featural and spatiotemporal information. Neuropsychologia 47, 657–662. 10.1016/j.neuropsychologia.2008.11.014 19071143 PMC2705971

[B77] Wilcox T. Bortfeld H. Woods R. Wruck E. Boas D. A. (2005). Using near- infrared spectroscopy to assess neural activation during object processing in infants. J. Biomed. Opt. 10, 11010. 10.1117/1.1852551 15847576 PMC1769348

[B78] Wilcox T. Bortfeld H. Woods R. Wruck E. Boas D. A. (2008). Hemodynamic response to featural changes in the occipital and inferior temporal cortex in infants: a preliminary methodological exploration. Dev. Sci. 11, 361–370. 10.1111/j.1467-7687.2008.00681.x 18466370 PMC2584206

[B79] Wilcox T. Haslup J. A. Boas D. A. (2010). Dissociation of processing of featural and spatiotemporal information in the infant cortex. NeuroImage 53, 1256–1263. 10.1016/j.neuroimage.2010.06.064 20603218 PMC2950116

[B80] Wilcox T. Hirshkowitz A. Hawkins L. Boas D. A. (2014). The effect of color priming on infant brain and behavior. NeuroImage 85, 302–313. 10.1016/j.neuroimage.2013.08.045 24007805 PMC3871198

[B81] Wilcox T. Stubbs J. Hirshkowitz A. Boas D. A. (2012). Functional activation of the infant cortex during object processing. NeuroImage 62, 1833–1840. 10.1016/j.neuroimage.2012.05.039 22634218 PMC3457789

[B82] Zosh J. M. Feigenson L. (2012). Memory load affects object individuation in 18-month-old infants. J. Exp. Child Psychol. 113, 322–336. 10.1016/j.jecp.2012.07.005 22932473

